# Aldehyde Dehydrogenase 1 in Gastric Cancer

**DOI:** 10.1155/2022/5734549

**Published:** 2022-03-09

**Authors:** Ling Wang, Luosha Wang, Yang Yu, Ruiliang Su, Yating Zhang, Yumin Li, Mingde Zang

**Affiliations:** ^1^Department of Gastric Cancer Surgery, Fudan University Shanghai Cancer Center, Shanghai, China; ^2^Department of Oncology, Shanghai Medical College, Fudan University, Shanghai 200032, China; ^3^Lanzhou University Second Hospital, Lanzhou, Gansu, China; ^4^The Second Clinical Medical College of Lanzhou University, Lanzhou, Gansu, China

## Abstract

Gastric cancer (GC) is a disease that threatens human health. It is thus crucial to clarify the mechanisms involved in GC development and discover diagnostic biomarkers and therapeutics. As a cancer stem cell marker, aldehyde dehydrogenase 1 (ALDH1) is involved in the development, progression, and treatment of GC. This review evaluated the prognostic value of ALDH1 and explored its mechanism of action in GC. Importantly, ALDH1 is an informative biomarker in clinical practice as it has specific relationships with indicators, such as metastasis and overall survival. Additionally, ALDH1 interacts with genes and exhibits properties that mimic stem cell characteristics amongst other mechanisms employed in the occurrence and progression of GC. Our results, therefore, provide evidence of possible clinical utility of ALDH1 as a GC therapeutic target.

## 1. Introduction

Gastric cancer (GC) ranks fifth in morbidity and is the third leading cause of death, with 1.0 million diagnosed cases and 783,000 deaths worldwide in 2018 [1]. The incidence of GC is high in East Asia, South America, and the Soviet region, and China has the highest incidence [1]. Moreover, China was ranked second and third for morbidity (29.31/100,000 persons) and mortality (21.16/100,000 persons) in 2015, respectively [2]. In particular, Gansu province has the highest incidence and mortality of GC at 62.37/10,000 persons and 46.27/10,000 persons, respectively [3]. Although there is progress in surgical techniques, chemotherapeutic regimens, radiation, and targeted therapies, the clinical outcomes of patients with GC remain poor. The diagnostic rate of GC in its early stages is low because of the lack of specific symptoms, and diagnosis of advanced GC has limited clinical benefits [4]. In a late-stage unresectable or metastatic disease, which affects approximately 80% of patients, the 5-year survival rate is <5% [5]. Therefore, it is important to elucidate GC pathogenesis and discover diagnostic biomarkers and therapeutic approaches for GC.

Cancer stem cells (CSCs) are involved in the self-renewal, asymmetrical division, differentiation, progression, and resistance of cancer cells [6, 7]. Notably, differentially expressed markers are used to authenticate CSCs [8]. CSCs are relevant in chemotherapy resistance, metastasis, recurrence, and tumor immune microenvironment in GC [9]. Aldehyde dehydrogenases (ALDHs), which can be classified into 19 different human ALDH isozymes, are metabolic stem cell markers. ALDHs participate in betaine biosynthesis of retinoic acid and *γ*-aminobutyric acid, which regulate cellular homeostasis. In addition, ALDHs also oxidize a series of endogenous and exogenous aldehydes to carboxylic acids, protecting them from oxidative stress in living organisms. In stem and progenitor cells, ALDH is derived from normal tissues and its expression is enhanced. Of note, ALDHs are involved in clone growth, self-renewal, drug resistance, and tumor-initiating capacity [10].

As a vital stem-like cell marker, ALDH1 is involved in the oxidation and detoxification of malignant tumors [11]. Although the functions of different ALDH1 isoforms contribute to ALDH1 activity, ALDH1A1 plays a dominant role [12]. In various cancers, as ALDH1 expression increases, self-renewal, proliferation, and carcinogenesis also increase [13, 14]. Of note, ALDH1 is an inhibitor of disulfiram, a well-known drug used to treat alcohol abuse which also has antitumor effects. Thus, hypothetically, it can be utilized in patients with GC in the future [15]. Many studies have verified that ALDH1 is a potential prognostic biomarker for colorectal, lung, and breast cancers [14]. In recent years, the expression of ALDH1 has been detected in GC; therefore, we reviewed the significance of ALDH expression in GC.

## 2. Clinical Value of ALDH1 in GC

Since 2012, many researchers worldwide have focused on the relationship between ALDH1 expression and GC. In multiple retrospective studies, patients who were pathologically diagnosed with GC but were not receiving radiotherapy or chemotherapy were selected, and gastritis and para-cancer tissues were used as controls. In GC, para-cancer, and noncancerous tissues, the expression of ALDH1 was measured by immunohistochemistry. Different studies included approximately 36–1072 GC cases, and ALDH1 was expressed in 43.9%–100% of cases [16–19]. ALDH1 positivity ranged from 5.77% to 24.1% and 10%–43.4% in noncancerous and para-cancer tissues, respectively [19–21]. ALDH1A1 positivity, respectively, ranged from 43.2% to 89.4% and 10%–43.4% in cancer tissue and in para-cancer tissues [16–21]. In most studies, noncancer tissues or para-cancer tissues were used as the control group. Yang et al. compared the expression of ALDH1 in cancer tissues, para-cancer tissues, and noncancer tissues (43.9%, 10.2%, and 5.77%, respectively) [18]. Therefore, ALDH1 expression was higher in GC tissues than in the noncancer and para-cancer tissues.

Senel et al. found no statistically significant relationship between the expression of ALDH1 and clinicopathologic parameters, recurrence-free survival, or overall survival (OS) [22]. In addition, Yang reported that ALDH1 expression is not correlated with age; sex; tumor, node, metastasis (TNM) stage; Lauren classification; or survival rates in GC [23]. Wakamatsu et al. reported that ALDH1 is related to an advanced T stage, TNM stage, intestinal histology, and a poor 5-year OS [24]. Additionally, Wu et al. found that ALDH1 is associated with age, depth of gastric wall invasion, differentiation, lymph node metastasis, and median OS [17]. Furthermore, Zhang et al. found that ALDH1 is related to lymph node metastasis, tumor differentiation, tumor pTNM stage, and 5-year OS [19]. Although there is a contradiction between the results of these studies, most of the findings are consistent. Therefore, ALDH1 may be a useful diagnostic and prognostic marker for GC.

## 3. ALDH1 Mechanisms in GC

The pathogenesis of GC is overly complex and may be related to *Helicobacter pylori* infection, oncogenes, tumor suppressor genes, abnormal differentiation, unnatural cell cycle regulation, and abnormal signaling pathways [25]. Notably, ALDH1A1 also participates in the occurrence and development of GC.

### 3.1. ALDH1 and *H. pylori*

Mao et al. concluded that ALDH1 is positively correlated with *H. pylori* infection. ALDH1 expression and the abundance of *Helicobacter pylori* were significantly increased in GC tissues than in normal gastric mucosa [26]. Zhao reported that ALDH1 protein expression was increased in the *H. pylori*-positive cases compared to the *H. pylori*-negative cases and that there was a positive correlation between ALDH1 expression and positive *H. pylori* staining. Therefore, *H. pylori* may be associated with the upregulation of ALDH1 [27]. In *H. pylori*-associated gastritis, the number of ALDH1-positive cells increased and contributed to the progression of neoplasia in the gastric mucosa. The age-related increase in cancer stem/stem-like cells in the gastric mucosa may explain the increased incidence of GC during aging [28].

### 3.2. Stem Cell Properties of ALDH1 in GC

Zhang reported that the positive rate of ALDH1 expression was 3.21%, 2.76%, and 1.89% in MKN-45, SGC7901, and MKN28 gastric carcinoma cells, respectively [20]. Wang et al. stated that the positive expression ratio of ALDH1 in MGC-803, BGC-823, and MKN-45 cells was 13.00 ± 1.34%, 5.80 ± 2.15%, and 36.50 ± 5.40%, respectively [29]. Moreover, ALDH1 expression was enhanced in MKN-45 and SGC7901 sphere cells than in typical MKN-45 and SGC7901 cells [30]. The expression of ALDH1 was higher in the peritoneal multicellular aggregates/spheroids of exfoliated GC cells than in scattered-free cancer cells. Multicellular aggregates showed increased efficiency in colony and tumor sphere formation, ALDH1 levels, drug resistance against 5-fluorouracil and oxaliplatin, and number of xenograft tumors. In the ALDH1^+^ cell group, the colony/tumor formation rates in mice were higher, and the sensitivity to 5-fluorouracil was lower [29, 31].

Overexpression of ALDH1A1 in MKN-28, a GC cell line, by the recombinant plasmid, pEGFP-N1-ALDH1A1, can increase the proliferation, colony formation, and invasion ability of cancer cells [32, 33]. In patients with GC and ascites, the level of ALDH1 was significantly higher than that in patients with a benign disease. The high expression of ALDH1 in GC cells can assist them in escaping the killing effect of macrophages by antagonizing tumor necrosis factor alpha and other effector molecules secreted by macrophages, resulting in increased proliferation and invasion capacity [34]. The high ALDH1 expression in GC cells (SGC7901) improves stem cell characteristics and antagonizes the effects of macrophages, thus affecting the cells' survival rate, anti-apoptosis, invasion, migration, and cloning abilities.

Using small interfering RNA interference, the expression of ALDH1A1 was downregulated in the GC cell line MKN-45. ALDH1A1 can inhibit the proliferation, colony formation, and invasion abilities of these cells and downregulate the expression of matrix metallopeptidases 2 and 9, Wnt1, Wnt5, and *ß*-catenin in MKN-45 cells. Importantly, ALDH1A1 silencing can decrease cell viability and inhibit cell migration and invasion via regulation of the Wnt signaling pathway in MKN-45 cells [35, 36].

These results support that, in GC, ALDH1+ cells have stem cell properties, and the growth of GC cells is repressed on inhibition of ALDH1 expression.

### 3.3. ALDH1 Interacts with Genes in GC

Studies on GC have focused on abnormal cell energy metabolism, self-sufficient growth signals, genomic instability and mutation, insensitivity to anti-growth signals, avoidance of immune surveillance, tissue invasion and metastasis, resistance to cell death, continuous angiogenesis, unlimited replication capacity, and promotion of tumor inflammation [37]. Many genes, proteins, and signaling pathways are involved in these processes.

Ror*β*, a hormone receptor in *Choristoneura*, is upregulated and promotes cell differentiation in GC, and in one study, ALDH1 and Wnt pathways were inhibited when Ror*β* was highly expressed in 200 GC tissues [38]. Moreover, cancer testis 45 antigen A1 is upregulated in GC tissues, and its overexpression upregulates the expression of ALDH1A3 in HGC-27 cells and promotes the development and metastasis of GC [39]. Furthermore, high mobility group protein A2 (HMGA2) is upregulated in GC and is related to poor prognosis. In one study, ALDH1 expression was increased in HMGA2-overexpressing GC stem cells (MKN-74 and MKN-28) but was decreased in human GC cells (MKN-45 and MGC803 cells) when HMGA2 was knocked down. Of note, HMGA2 promotes the self-renewal of GCSCs [40]. Progestin and adipoQ receptor 3 is downregulated and negatively related to tumor size, stage, and OS in GC, and its overexpression in BGC-823 cells leads to decreased ALDH1A1 expression [41]. A transcriptional coactivator with a PDZ-binding motif (TAZ) is upregulated in GC and is related to an advanced TNM stage and poor prognosis. Overexpression of TAZ upregulated the expression of ALDH1 in MKN28 and MGC803 cells, suggesting that the expression of TAZ can promote the occurrence of epithelial to mesenchymal transition and cancer stem cell and then promote the formation of vasculogenic mimicry [42].

Bromodomain and extra-terminal domain protein 4 (BRD4) was dramatically augmented in GC tissues compared to normal adjacent tissues [43]. One study reported that BRD4 was negatively correlated with OS and first and postprogression survival of patients with GC. BRD4 was knocked down in MGC-803 and BGC-823 GC cells, and the expression and activity of ALDH1 were significantly decreased. It also inhibited Wnt/*β*-catenin signaling by attenuating miR-216a-3p and promoted the stemness of GC cells [43]. Testis-associated highly conserved oncogenic long noncoding RNA was significantly increased and related to stemness markers in GC and when it was stably knocked out in MKN-45 cells, ALDH1 expression and globule formation capacity were decreased [44]. Silencing miR-95 or overexpressing the miR‐95 inhibitor (dual-specificity phosphatase 5) suppressed ALDH1 expression and tumor sphere formation in GC cells (BGC803), and GC development was inhibited via the dual-specificity phosphatase 5‐dependent mitogen-activated protein kinase pathway mediated by miR‐95 knockdown [45]. miR-625 silencing resulted in increased ALDH1A1 expression and luciferase activity of the wild-type ALDH1A1 construct. In SGC7901/adriamycin amycin cells and SGC7901/vincristine cells, the downregulation of miR-625 elevated the mRNA and protein levels of ALDH1A1. Therefore, ALDH1A1 may be a direct target of miR-625. When ALDH1 was depleted, the miR-625 silencing-increased IC50 values for the four tested chemotherapeutic agents (adriamycin amycin, vincristine, 5 fluorouracil, and cis-dichlorodiammine platinum) were reversed. In contrast, when ALDH1A1 was overexpressed, the reduced resistance to multiple drugs caused by miR-625 overexpression was mainly resumed. Hence, ALDH1A1 is a crucial target of miR-625-associated reversal of this multidrug resistance in GC cells [46]. ALDH1+ cells were able to self‐renew and generate heterogeneous cell populations and were more tumorigenic than ALDH1− cells in diffuse-type gastric carcinoma cells. In human diffuse-type gastric carcinoma cells (OCUM‐2MLN and HSC‐39), regenerating islet‐derived family member 4 (REG4) is upregulated in ALDH1+ cancer-initiating cells. Transforming growth factor beta downregulated REG4 and ALDH1 expression, which is related to the decline in cancer-initiating cell population size and tumorigenicity. It was concluded that the enhanced tumorigenic ability of ALDH1+ cells depended on REG4 [47]. Taken together, as a stem cell marker, ALDH1 is involved in the regulation of various genes and proteins in the occurrence and development of GC.

### 3.4. ALDH1 and GC Therapy

Surgical treatment after early diagnosis of GC has a good prognosis, but most GCs are diagnosed in the middle and late stages, and the opportunity for surgery is lost. Chemotherapy is currently the predominant therapy of advanced GC; however, there are patients with poor sensitivity to existing chemotherapy drugs. Therefore, it is necessary to identify new therapeutic drugs and treatment regimens that can be used clinically.

In MKN-45 tumor spheres, approximately 35% of cells expressed ALDH1, and this percentage was significantly suppressed gradually from 2 to 5 days of all-trans retinoic acid treatment. In diffuse and intestinal types of gastric carcinoma, all-trans retinoic acid appears to repress tumor growth and, more importantly, target GCSCs [48]. Verteporfin weakened the CSC pool of GC cell lines and interfered with their capacity to grow into tumor spheres *in vitro*. The number of ALDH+ cells dramatically reduced with 1 *μ*M verteporfin treatment in MKN-45 tumor spheres [49]. In advanced GC, Xiao-ai-ping combined with intravenous chemotherapy could more efficiently kill cancer cells and inhibit cancer cell proliferation and invasion. ALDH1 expression was significantly lower in the GC tissues from the Xiao-ai-ping group [50]. GC cells (NCI–N87 and SNU-1) with a high expression of ALDH1 exhibited increased CSC characteristics, colony formation, floating spheroid bodies, and resistance to conventional chemotherapeutic 5 fluorouracil and cis-dichlorodiammine platinum. Salinomycin efficiently killed ALDH stem-like cells in GC [51]. 7-Difluoromethoxyl-5,4′-di-n-octyl genistein restrained the stem-like characteristics of GC cells and reversed the epithelial-mesenchymal transition phenotype by modulating FoxM1 and Twist1 expressions. FoxM1 siRNA transfection cooperated with 7-difluoromethoxyl-5,4′-di-n-octyl genistein inhibited cell migration, self-renewal capacity, cell invasion, and the expression of ALDH1 [52]. Taken together, drugs inhibit the growth of GC and reverse drug resistance by repressing the expression of ALDH1; thus, ALDH1 may be a therapeutic target.

## 4. Conclusions and Prospects

ALDH1 is highly expressed in GC tissues and cell lines and may be related to differentiation, TNM stage, prognosis, cell growth, and invasion. As a CSC marker, the expression of ALDH1 is inhibited by nucleotides, proteins, or drugs, leading to the inhibition of GC cell growth by inhibiting the characteristics of GC stem cells. ALDH1 inhibitors may also be used in the clinical treatment of GC. Therefore, ALDH1 can be used as a therapeutic target to evaluate the prognosis of GC.
